# The Accuracy of Classification Systems in Nonsyndromic Sagittal Craniosynostosis

**DOI:** 10.1097/SCS.0000000000009670

**Published:** 2023-08-28

**Authors:** Tymon Skadorwa, Joanna Skadorwa, Olga Wierzbieniec

**Affiliations:** * Department of Pediatric Neurosurgery, Bogdanowicz Memorial Hospital for Children; † Department of Descriptive and Clinical Anatomy, The Medical University of Warsaw; ‡ Wolski Municipal Hospital, Warsaw, Poland

**Keywords:** Children, craniosynostoses, infants, sagittal synostosis, scaphocephaly

## Abstract

Numerous classification systems of nonsyndromic sagittal craniosynostosis (NSC) are applied but none has gained a wide acceptance, since each classification is focused on distinct aspects. The aim of the study was to assess the accuracy of 4 classifications of NSC discussed in the literature by defining the associations among the classifications, individual features (sex, age, cranial index), and objective morphologic criteria (frontal bossing, retrocoronal constriction, sagittal ridge, and occipital bulleting). The study was conducted on anonymized thin-cut CT scans of 133 children with NSC 1 to 12 months old (mean age 5.42 mo). The type of cranial dysmorphology was assessed using 4 classification systems, focusing on skull shape, pattern of sagittal suture closure (Heuzé classification), deformation of skull vault (Sakamoto classification), and a single-dominant feature (David classification). Each patient was also independently investigated for the presence of morphologic criteria. A multivariate analysis was performed to explore the relations among the classifications and assess their accuracy. In the analyzed cohort sphenocephaly (38.3%), CFF type by Heuzé (30.8%), type I by Sakamoto (72.9%), and a central type by David (42.9%) were dominant findings. Regarding the morphologic criteria, frontal bossing was observed the most frequently (91.7%). The age of patients and cranial index differed significantly among the shapes of skull and David classifications (*P*<0.001). The shape-based system showed the strongest correlation with other classifications and with measurable variables. Other classifications have much in common and some overlap, but none of them constitutes a standalone system to define all aspects of cranial dysmorphology in NSC.

Nonsyndromic sagittal craniosynostosis (NSC) is the most common type of premature closure of cranial sutures. A typical skull deformation includes an elongation of the skull with a reduction of its transverse dimension, a longitudinal sagittal ridge, frontal and occipital bossing, or retrocoronal depression. Despite well-described morphology, not all of these features occur simultaneously, which results in the fact that NSC patients are still a heterogeneous group, difficult to define with a single classification.^1,2^

Several terms are used to describe cranial dysmorphology in NSC. The term “dolichocephaly” has been associated with the elongation of the skull since the beginning of research on congenital cranial deformities. However, this original term, used by Virchow, has nowadays lost its pathological significance. The first association between morphologic alterations of the skull and face and premature closure of cranial sutures was given by Virchow,^3^ although it was Sear in 1937 who first introduced the term “craniosynostosis”.^4^ The term “scaphocephaly” appears in the classification in the study by Simmons and Peyton (1947),^5^ which extended the description in the study by Greig (1926)^6^ for oxycephaly to include new categories linking the “keel-shaped skull” with premature closure of the sagittal suture. This system was simplified in the study by Fairman and Horrax^7^ in 1949 who divided craniosynostosis into 2 main groups: incomplete and complete synostoses. Scaphocephaly has been classified as “incomplete craniosynostosis” with 4 morphologic variations, resulting from partial and/or progressive involvement of sagittal suture (leptocephaly, clinocephaly, sphenocephaly, bathrocephaly). In the work from 1958, Bertelsen introduced three other categories of cranial synostoses: simple craniosynostoses, craniofacial dysostoses, and acrocephalosyndactylies.^8^ Scaphocephaly was kept in the group of simple craniosynostoses from the classification in the study by Montaut and Stricker^9^ in 1977 and classified as a “primary craniosynostosis” in the Duggan^10^ classification from 1970. Recent classifications also included genetic aspects,^11^ determined the degree of premature closure of the sagittal suture,^12^ focused on the deformation mechanism,^13^ type of cranial dysmorphology ^14^ or described single dominant morphologic features.^15^

Nonsyndromic NSC, despite being the most commonly diagnosed and studied congenital cranial deformation, still lacks a concise classification system. In May 2022, a new European call for investigation and collaboration has been announced^16^ but we feel that in the matter of classification, there still exist inaccuracies, preventing effective research in this field.

Numerous distinct classifications are in use nowadays but none of them covers all the morphologic features of NSC described in the literature. Therefore, the aim of this study was to define the associations between the most commonly used classifications and individual characteristics such as sex, age, and cranial index. The second goal of this work was to assess the accuracy of these classification systems and to establish the associations between them and objective morphologic criteria, such as frontal bossing, retrocoronal constriction, sagittal ridge, and occipital bulleting.

## MATERIALS AND METHODS

### Study Design

This retrospective study was performed on anonymized digital data. No human subject was directly involved and consent to participate was not required by the protocol. All patients consented to collect the medical data in writing. This retrospective study was approved by the institutional bioethics committee (decision number AKBE/110/2021), and abides by the 1964 Helsinki Declaration and its later amendments or comparable ethical standards.

### Population

The group of patients with NSC 1 to 12 months old included 133 consecutive children treated in our institution between 2010 and 2021. All patients had preoperative CT scans. Mean age of the studied group was 5.42 months, median age 5.03 months, and SD 2.49. Sex ratio (M:F) was 2.91.

## METHODS

The type of cranial deformation was investigated using preoperative thin-cut CT scans provided with Siemens Somatom Emotion with parameters: slice thickness 0.5 mm; the exposition was performed with source voltage 270 kV and current of 100 mA. All scans were analyzed with software provided by the manufacturer (INFINITT PACS, INFINITT Healthcare Co., Ltd., 2016).

**FIGURE 1:**
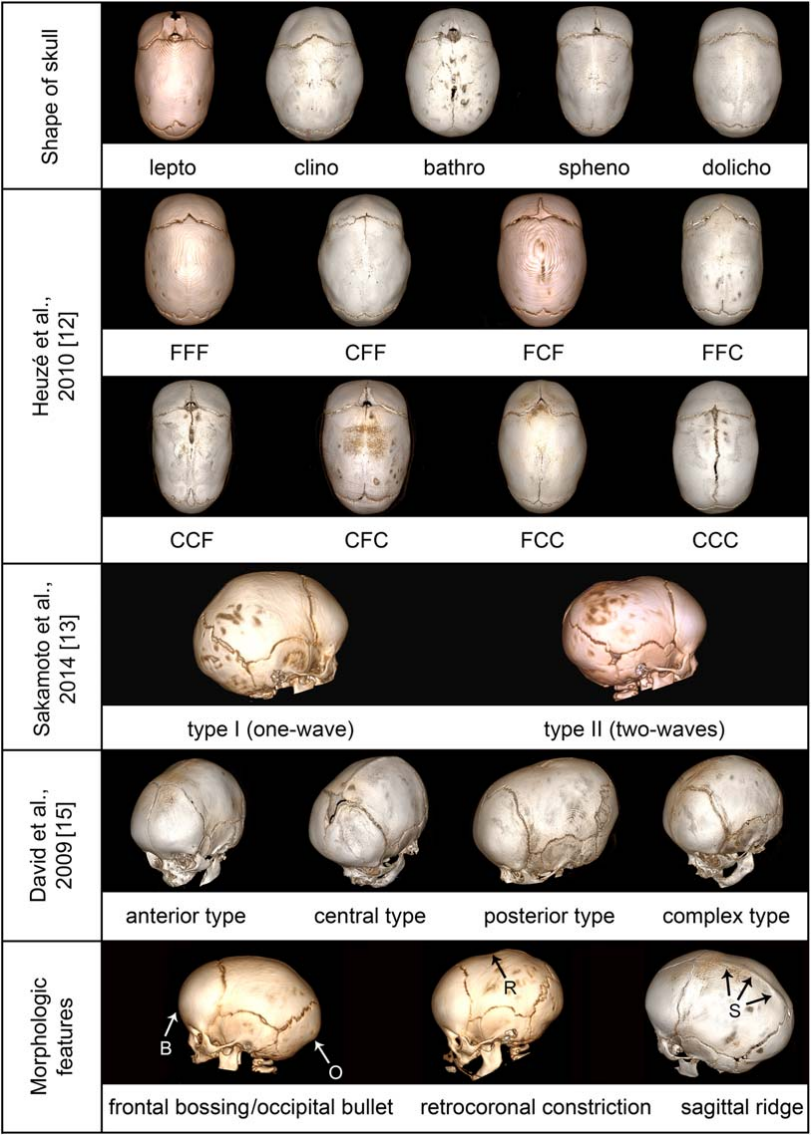
An overview of the classifications and morphologic features of nonsyndromic sagittal craniosynostosis (NSC) used in the study.

Two arms of evaluation were designed to assess the accuracy of classification systems. In the first arm, the patients were assigned to groups according to 4 distinct classifications (Fig. 1).

1. Shape of the skull—Di Rocco et al^1^ provided 5 main types of cranial deformation in sagittal synostosis, adapted from classical anatomical descriptions.^7,11^ Four of them were reported to result from a partial closure of the sagittal suture and included: leptocephaly (equal narrowing of the skull), clinocephaly (with retrocoronal depression), bathrocephaly (with prominent, bulging occiput) and sphenocephaly (with the prominence of bregma and forehead width exceeding the interparietal diameter). The fifth type was dolichocephaly, reported to result from a complete fusion of the sagittal suture. We adopted this classification system with 1 modification: the category “dolichocephaly” was assigned to all undetermined cases (no single characteristic feature as in other 4 types or a combination of more than 1 feature) and it was not necessarily associated with the complete fusion of the sagittal suture.
2. The second classification was given by Heuzé et al^12^. In their study, a pattern of sagittal suture fusion (F) or closure (C) was determined using specific codes for every 1 of 3 portions of the sagittal suture (ie, type “CFC” meaning the closure of anterior and posterior thirds, and a fusion of the middle third portion of the suture; see Fig. 1).
3. Third classification was provided in the study by Sakamoto et al.^13^ who distinguished 2 types of cranial vault deformity: type I (1-wave) and type II (2-wave) (Fig. 1).
4. The fourth classification was proposed in the study by David et al.^15^ and included 4 categories, based on the presence of a single dominant feature: anterior type—with a transverse retrocoronal band, central type—with a markedly heaped-up sagittal suture, posterior type—when a prominent occiput was present, and a complex type—with no single dominant characteristic.

The categories were assigned by 2 researchers independently with an inter-rater reliability of 81.6% (kappa 0.792; 95% confidence interval, 0.723–0.877; *P*<0.01). When the choices of the 2 observers did not correspond, the researchers discussed them before the final distribution to reach a consensus and avoid observational bias.

In the second arm of the study, the third researcher independently evaluated the CT scans in search of the presence of typical morphologic features, discussed in the articles by Schmelzer et al.^14^ and by Diab et al^2^: frontal bossing (B), retrocoronal constriction (R), occipital bulleting (O), and sagittal ridge (S) (Fig. 1). In this part of the evaluation no classifications were used but pure morphologic criteria to match them with particular categories used in the first arm of the analysis.

After assigning the categories a multivariate analysis was performed to explore the relations between the classifications, the effects of matching them with morphologic criteria and to assess the accuracy of classifications. Individual characteristics (sex, age, cranial index) were compared between the classifications. Cranial index (CI) was defined by the relation of a maximal transverse dimension of the skull to a maximal longitudinal dimension taken at the same level in the horizontal plane.

### Statistical Analysis

The statistical analysis was performed with TIBCO Data Science/Statistica software by StatSoft Europe, version 13.3 PL for Microsoft Windows 10 Pro. The differences between the variables were assessed with the chi-squared test for categorical variables and nonparametric Mann-Whitney-Wilcoxon rank-sum test or non-parametric ANOVA (Kruskal-Wallis test) for quantitative data. Effect sizes were measured with Spearman’s rho, Pearson’s C or Cramer’s V. Probability values below 0.05 were considered statistically significant.

## RESULTS

### First Arm of Evaluation

The studied group consisted of 99 boys (74.4%) and 34 girls (25.6%). A distribution to classification groups revealed that sphenocephaly (51/133 cases) was a dominant skull shape (38.3%) followed by clinocephaly and bathrocephaly. Regarding the Heuzé classification, a CFF type, representing the fusion of a middle and posterior portions of the sagittal suture was the most common pattern across the entire group, found in 41 of 133 cases (30.8%). Other common patterns included the fusion of the whole suture (FFF), anterior and middle (FFC), or just its middle portion (CFC). The remaining patterns (FCC, CCF, FCF, CCC) were found in less than 5% of the cohort and therefore were not further analyzed. In terms of Sakamoto classification, 1-wave type was found present in 97 of 133 patients (72.9%) and for the David classification, the central type was noted the most frequently (57/133 cases, 42.9%). Detailed numbers of patients categorized by investigated classification systems are presented in Table 1 (as shown in Supplementary Digital Content 1, http://links.lww.com/SCS/F397).

### Second Arm of Evaluation

Frontal bossing was a dominant morphologic feature and was present in 122 of 133 cases (91.7%). Retrocoronal constriction was identified in 80 of 133 cases (60.2%), occipital bullet in 91 of 133 cases (68.4%), and sagittal ridge in 89 of 133 cases (66.9%). A distribution of morphologic features by categories is presented in Table 2 (as shown in Supplementary Digital Content 1, http://links.lww.com/SCS/F397). Subsequently, each category was assigned a morphologic code, that included a designed letter (B, R, O, S) when a corresponding feature was present in more than 2 of 3 of cases (66.6%).

### Multivariate Analysis

Obtained results were analyzed statistically in terms of possible associations. No significant correlation between sex and the classifications was found.

The age differed significantly among the shapes of skull (H=23.6770, *P*<0.0001), Heuzé (H=39.3338, *P*<0.0001), and David classifications (H=12.7508, *P*=0.0052). Mean age was the lowest in sphenocephaly, CFC, and central type by David and the highest in leptocephaly, FFF, and anterior type by David (as shown in Supplementary Digital Content 1, Table 3, http://links.lww.com/SCS/F397). The Sakamoto classification seemed not to be influenced by age, as age did not significantly differ between the 1-wave and 2-wave groups. The age significantly correlated with Heuzé and David classifications and with the skull shape.

Cranial index was significantly different among skull shapes (*H*=29.2667, *P*<0.001) and the categories of David classification (*H*=29.5602, *P*<0.001). CI revealed a positive correlation with skull shape (Spearman rho 0.27, *P*=0.0021).

The prevalence of morphologic features, assessed as a second arm of the study, did not significantly correlate with the classification systems. The correlations between the classifications, cranial index, sex, and age are presented in Table 4 (as shown in Supplementary Digital Content 1, http://links.lww.com/SCS/F397).

**FIGURE 2:**
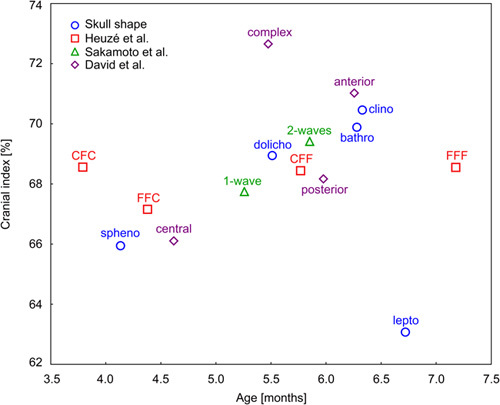
A scatterplot of CI against age showing mean values of CI and age for each category.

The relations between the categories of studied classification systems are presented in the scatterplot of CI against age showing mean values of CI and age for each category (Fig. 2).

## DISCUSSION

The heterogeneity of NSC has been demonstrated in many studies.^1,17^ In addition, new articles, analyzing the morphology of selected subtypes are appearing in the literature.^18^ The presence of distinct classification systems highlights this concept, as each of them brings unique radiologic and morphologic criteria to the characteristics of NSC. In addition, apart from the purely morphologic factors, new criteria are incorporated into the description of NSC, such as the alterations of cerebrospinal fluid spaces, discussed in several studies since the late 1980s of XXth century^19–23^ and used in current attempts to systematize the radiomorphologic determinants of NSC.^2^ Novel approaches aim to characterize NSC morphology in yet other ways—through three-dimensional measurements of the skull or computer simulations of its growth.^24,25^ The genetic factor responsible for the diversity of NSC is also sought,^26^ and the morphology itself is used for the evaluation of aesthetic^27–29^ and functional outcomes.^30,31^

Regarding the tendency to systematize the NSC morphology, we feel that the number of definitions and terms used in the literature may be confusing, that is, the term “scaphocephaly” refers to all types of deformations caused by premature closure of the sagittal suture, but the term “dolichocephaly,” formerly used for description of cranial deformities, does not strictly mean pathology, but a variant of a normal skull, characterized by the predominance of the longitudinal dimension over the transverse dimension.^32^ The study by Di Rocco et al described different subtypes of sagittal synostosis by listing morphologic variations in relation to an obliterated portion of sagittal suture. According to that article, dolichocephaly would result from a complete fusion of the suture, whereas partial fusion would give 4 other types of cranial dysmorphology. In our study, we used a modified system adapted from the article by Di Rocco et al^1^ assigning the term “dolichocephaly” to each case of NSC other than the remaining 4 types, but not necessarily present with a complete fusion of the suture, as in our population only 5 of 12 of dolichocephaly patients showed the FFF pattern. Other cranial shapes in our material neither corresponded exclusively with the reported pattern of suture closure.

The work by Heuzé et al^12^ explains more about this topic. The authors showed that the sequence of suture obliteration includes the following irreversible stages: patency, closure (suture visible as a fine line), and fusion (no evidence of suture between 2 parietal bones). These stages occur in a specific order, which by definition precludes the transition from the “F” phase to the “C” phase and makes the patterns with more fused portions to be seen in older age. Heuzé et al examined 43 NSC patients 0.9 to 9.0 months old. Patterns CCC and FCC were the least frequently observed. The most common pattern was FFF (37.2%, mean age 5.8 mo), followed by CFC (23.3%), FFC (18.6%), and CFF (16.3%). The distribution of patterns across our population was different but we also observed a dependence on age (mean age of children presenting the CFC pattern was 3.79 mo, the FFC pattern 4.38 mo, the CFF pattern 5.77 mo, and the FFF pattern–7.18 mo), which to our opinion supports the idea of a fusion continuum proposed in the article by Heuzé et al. What is more, some patterns of sagittal suture fusion observed in NSC patients correspond more likely with specific skull shapes (V=0.38, *P*<0.001), that is, CFC with sphenocephaly, CFF with bathrocephaly, or FFF with clinocephaly (Fig. 2). However, the dependence of the skull shape on the pattern is not absolute and individuals presenting the same pattern may reveal morphologic dissimilarities.^33^

The Sakamoto classification brings a new perspective to this matter.^13^ The authors examined 18 patients 0 to 29 months old, assigning type I deformation (1-wave) to 9 patients, and type II (2-wave) to another 9. They noticed that type I was associated with a partial closure of the sagittal suture (posterior fusion) and type II with a total fusion. The mean age for type I deformation was 9.1 months and for type II deformation 10.9 months. In our material, 1-wave type was predominant in individuals with sphenocephaly, clinocephaly, dolichocephaly, and leptocephaly. Only 2-wave type showed a characteristic affinity for bathrocephaly (C=0.63, *P*<0.0001). Compared to the suture closure pattern, we did not observe so significant correlations: 1-wave was most often observed in combination with CFF, FFF, and CFC patterns, and 2-wave with CFC, FFC, and FFF patterns. Therefore, in both types of deformation, we observed both complete and partial fusion of the sagittal suture (C=0.36, *P* = 0.0061). Similarly to skull shape, the Sakamoto system showed a high correlation with David classification (C=0.57, *P*<0.0001), where 2-wave usually denoted posterior type (>70%) and 1-wave was associated with central or anterior types in over 80% of cases.

The classification by David et al^15^ is also related to cranial vault morphology, but it refers to the presence of a single dominant morphologic characteristic. The authors investigated CT scans of 55 infants with NSC 0.06 to 72 months old. The posterior type was the most commonly observed (35%), followed by central (29%), anterior (24%), and complex (13%) types. They concluded that 87% of scaphocephalic skulls fall into 3 distinct classes, defined by the presence of 1 dominating feature. Comparing to our findings, this classification seems to be the most difficult to reproduce, which was admitted in other articles by the authors themselves.^34^ Our study confirmed the predominance of each of the examined features in a different category of this system - retrocoronal constriction (R) in anterior type, sagittal ridge (S) in central type, and occipital bullet (O) in posterior type. David classification, however, did not take into account the occurrence of frontal bossing, which in our population was predominant in all categories and therefore should not be considered a differentiating element of the skull morphology in NSC patients.

Comparing the morphologic criteria with particular classifications allows us to notice certain regularities related to the definitions of individual categories. For example, the presence of an occipital bullet, typical in bathrocephaly, in our population was also a significant criterion in the posterior type by David and type II (2-wave) by Sakamoto. Matching these criteria with classifications shows that the same sets of features are described by different categories from distinct classification systems, that is, the combination “BOS” is typical not only for sphenocephaly, but also for the CFC pattern by Heuzé, whereas the “BRS” combination better characterizes clinocephaly and the FFF pattern, etc (Supplementary Digital Content, Table 2, http://links.lww.com/SCS/F397).

Meanwhile, regarding the correlation between classifications (Supplementary Digital Content, Table 4, http://links.lww.com/SCS/F397) and similarities in objective variables (Fig. 2), it seems that particular categories might be grouped into specific clusters, close in terms of mean age and CI. The group with the lowest means are patients classified as central type by David, sphenocephaly by shape, and presenting the CFC or FFC pattern by Heuzé. The second characteristic group is patients referred to as bathrocephaly, type II (2-wave) by Sakamoto, posterior type by David, and CFF pattern by Heuzé. Another group would be the clinocephaly-FFF-anterior type by David, occupying the rightmost area of the chart. An indistinct group consists of type I by Sakamoto (1-wave) and dolichocephaly, located mostly in the middle of the chart. Outsiders occupying the peripheral regions of the chart and characterized by the highest mean age or CI, include patients with leptocephaly and the complex type by David, which is probably due to the small representation of these groups in the analyzed cohort (Fig. 2).

It seems, therefore, that described groups, similar one to another in terms of mean age, CI, and morphologic criteria, actually belong to the same morphologic subtypes, although defined by different classification systems. Considering the strongest correlation with the shape of the skull, we propose to define these groups by shape. In addition, considering that frontal bossing as the most common feature in NSC is the least differentiating, it seems that it is not the bossing that determines a group allocation, but the next frequent morphologic feature, that is, sagittal ridge for sphenocephaly, retrocoronal constriction for clinocephaly and occipital bulleting for bathrocephaly.

Combining 2 arms of our analysis allowed us to assess the accuracy of investigated classification systems. The distribution of examined criteria confirms the legitimacy of separating the categories in the David classification—in our study, the percentage distribution of these features coincided with the idea of the authors of this system. However, the criteria differentiating individual categories in the David classification partially overlap with the features of morphology seen in a given skull shape (occipital bullet, retrocoronal depression), whereas others occur in different skull shapes or were not included in the classification (sagittal ridge, frontal bossing).

In turn, the Sakamoto classification is an attempt to explain the formation of the skull shape based on the degree of sagittal suture closure. This classification provides a unique view on the deformation of the vault, unheard of in other systems, but partially overlapping with the observed shapes, for example, sphenocephaly in 1-wave type. In our opinion, despite the convergence of some shapes, this system cannot be compared with others, because it contains too few categories and does not assess the occurrence of morphologic features as in the David system. In turn, the Heuzé classification does not refer to either the shape of the skull or the presence of morphologic features. It can therefore be concluded that each of the classifications describes a different aspect of NSC morphology, but they are not standalone systems. Our work is an attempt to bring these different aspects together as they share common parts that were clearly visible in both arms of our study.

## CONCLUSIONS

In this study, we managed to compare various NSC classification systems. As shown in the analysis, these classifications have much in common, and some overlap, but none of them constitutes a standalone system that defines all aspects of cranial dysmorphology in NSC. The shape-based system seems to be the most useful as it shows the strongest correlations with other classifications and with measurable variables. The classification by David et al to some extent duplicates these criteria and does not bring a genuine impact. The classification by Heuzé et al may be considered as a separate system, informing about the condition of the sagittal suture. It does not provide direct characteristics of skull dysmorphology but it allows, with some probability, to combine the pattern of suture closure with the shape of the skull. Finally, the Sakamoto classification does not create an independent system of grouping patients but clearly correlates with certain types of cranial deformation, completing the spectrum of morphologic diversity in NSC.

## Supplementary Material

SUPPLEMENTARY MATERIAL
