# Supplementary material for: The Accuracy of Classification Systems in Nonsyndromic Sagittal Craniosynostosis
Source: J Craniofac Surg. 2023 Aug 28;35(1):13–7. doi: 10.1097/SCS.0000000000009670 (PMC10749678; doi:10.1097/SCS.0000000000009670)
Supplement: SUPPLEMENTARY MATERIAL [file scs-35-013-s001.pdf]

Title: The accuracy of classification systems in nonsyndromic sagittal craniosynostosis.

Authors: Tymon Skadorwa, MD PhD , Joanna Skadorwa, MD, Olga Wierzbieniec, MD

Table 1

Clinical characteristics of the total cohort of NSC patients. *M* males, *F* females, *CI* cranial index, *SD* standard deviation.

		M (n=99)		F (n=34)		Total (n=133)	
Mean age ± SD (months)		5.30 ± 2.43		5.76 ± 2.65		5.42 ± 2.51	
Mean CI ± SD		68.46 ± 5.51		67.42 ± 4.69		68.21 ± 5.32	
		n	%	n	%	n	%
Shape of skull	sphenocephaly	38	28.6	13	9.8	51	38.3
	clinocephaly	25	18.8	14	10.5	39	29.3
	bathrocephaly	23	17.3	3	2.3	26	19.5
	dolichocephaly	9	6.8	3	2.3	12	9.0
	leptocephaly	4	3.0	1	0.8	5	3.8
Heuzé et al., 2010 [12]	CFF	25	18.8	16	12.0	41	30.8
	FFF	28	21.1	6	4.5	34	25.6
	CFC	27	20.3	6	4.5	33	24.8
	FFC	15	11.3	4	3.0	19	14.3
	FCC	2	1.5	1	0.8	3	2.3
	CCF	1	0.8	0	0.0	1	0.8
	FCF	0	0.0	1	0.8	1	0.8
	CCC	1	0.8	0	0.0	1	0.8
Sakamoto et al., 2014 [13]	type 1 (1-wave)	69	51.9	28	21.1	97	72.9
	type 2 (2-waves)	30	22.6	6	4.5	36	27.1
David et al., 2009 [15]	central type	44	33.1	13	9.8	57	42.9
	anterior type	22	16.5	11	8.3	33	24.8
	posterior type	24	18.0	7	5.3	31	23.3
	complex type	9	6.8	3	2.3	12	9.0

Table 2

Distribution of morphologic features by classifications. *B* frontal bossing, *R* retrocoronal constrction, *O* occipital bulleting, *S* sagittal ridge. A morphologic code for the whole category included a designed letter when a corresponding feature was present in more than 2/3 of cases.

		B	R	O	S	Code
Shape of skull	sphenocephaly	47	30	38	39	BOS
	clinocephaly	37	31	25	27	BRS
	bathrocephaly	22	6	18	13	BO
	leptocephaly	5	4	2	3	BR
	dolichocephaly	11	9	8	7	BRO
Heuzé et al., 2010 [12]	CFF	39	16	35	21	BO
	FFF	28	26	19	27	BRS
	CFC	31	21	24	28	BOS
	FFC	18	12	11	14	BS
Sakamoto et al., 2014 [13]	type 1 (1-wave)	90	69	64	71	BROS
	type 2 (2-waves)	32	11	27	18	BO
David et al., 2009 [15]	central type	52	33	32	52	BS
	anterior type	31	30	20	16	BR
	posterior type	28	9	29	11	BO
	complex type	11	8	10	10	BROS

Table 3

Mean age and CI by classifications. *CI* cranial index; *SD* standard deviation.

Classification system	Categories	Age [months]		CI [%]	
		mean	SD	mean	SD
Shape of skull	sphenocephaly	4.13	1.95	65.95	5.42
	dolichocephaly	5.51	2.43	68.94	6.17
	bathrocephaly	6.28	2.62	69.89	5.15
	clinocephaly	6.33	2.18	70.46	3.59
	leptocephaly	6.72	4.03	63.07	2.77
Heuzé et al., 2010 [12]	CFC	3.79	1.66	68.56	6.48
	FFC	4.38	1.57	67.16	6.42
	CFF	5.77	2.30	68.44	4.57
	FFF	7.18	2.61	68.55	4.33
Sakamoto et al., 2014 [13]	type 1 (1-wave)	5.26	2.51	67.75	5.35
	type 2 (2-waves)	5.85	2.40	69.42	5.14
David et al., 2009 [15]	central type	4.62	2.41	66.10	4.19
	complex type	5.48	2.59	72.66	7.45
	posterior type	5.98	2.50	68.17	5.44
	anterior type	6.26	2.24	71.02	3.41

Table 4

Correlations between the classifications. *NS* not significant.

	Sex	Cranial index	Age	David et al., 2009[15]	Sakamoto et al., 2014[13]	Heuzé et al., 2010[12]
Shape of skull	NS	ρ=0.2703 P=0.0021	r=0.3235 P=0.0002	V=0.6705 P<0.0001	C=0.6286 P<0.0001	V=0.3802 P<0.0001
Heuzé et al., 2010 [12]	NS	NS	r=0.2449 P=0.0048	V=0.3275 P=0.0040	C=0.3622 P=0.0060	x
Sakamoto et al., 2014 [13]	NS	NS	NS	C=0.5744 P<0.0001	x	
David et al., 2009 [15]	NS	NS	r=0.2561 P=0.0032	x		
Age	NS	NS	x			
Cranial index	NS	x				
